# Author Correction: The wide utility of rabbits as models of human diseases

**DOI:** 10.1038/s12276-019-0252-0

**Published:** 2019-07-04

**Authors:** Pedro J. Esteves, Joana Abrantes, Hanna-Mari Baldauf, Lbachir BenMohamed, Yuxing Chen, Neil Christensen, Javier González-Gallego, Lorenzo Giacani, Jiafen Hu, Gilla Kaplan, Oliver T. Keppler, Katherine L. Knight, Xiang-Peng Kong, Dennis K. Lanning, Jacques Le Pendu, Ana Lemos de Matos, Jia Liu, Shuying Liu, Ana M. Lopes, Shan Lu, Sheila Lukehart, Yukari C. Manabe, Fabiana Neves, Grant McFadden, Ruimin Pan, Xuwen Peng, Patricia de Sousa-Pereira, Ana Pinheiro, Masmudur Rahman, Natalie Ruvoën-Clouet, Selvakumar Subbian, Maria Jesús Tuñón, Wessel van der Loo, Michael Vaine, Laura E. Via, Shixia Wang, Rose Mage

**Affiliations:** 1 CIBIO, InBIO, Research Network in Biodiversity and Evolutionary Biology, Universidade do Porto, Campus de Vairão, Rua Padre Armando Quintas, 4485-661 Vairão, Portugal; 2 Departamento de Biologia, Faculdade de Ciências da Universidade do Porto, Rua do Campo Alegre, s/n, 4169-007 Porto, Portugal; 3 Instituto de Investigação e Formação Avançada em Ciências e Tecnologias da Saúde (CESPU), Gandra, Portugal; 4 Max von Pettenkofer Institute and Gene Center, Virology, National Reference Center for Retroviruses, Faculty of Medicine, LMU München, 81377 Munich, Germany; 5 Laboratory of Cellular and Molecular Immunology, Gavin Herbert Eye Institute, University of California, Irvine, School of Medicine, Irvine, CA 92697 USA; 6 Department of Molecular Biology and Biochemistry, University of California, Irvine School of Medicine, Irvine, CA 92697 USA; 7 Institute for Immunology, University of California, Irvine School of Irvine, School of Medicine, Irvine, CA 92697 USA; 8 Department of Medicine, University of Massachusetts Medical School, Worcester, MA 01605 USA; 9 Departments of Pathology, Microbiology and Immunology, and Comparative Medicine, Penn State University, Hershey, PA USA; 10 Institute of Biomedicine (IBIOMED) and Centro de Investigación Biomédica en Red de Enfermedades Hepáticas y Digestivas (CIBERehd), University of León, 24071 León, Spain; 11 Departments of Medicine and Global Health, University of Washington, Seattle, USA; 12 Bill and Melinda Gates Foundation, Seattle, WA USA; 13 Department of Microbiology and Immunology, Loyola University Chicago, Maywood, IL 60153 USA; 14 Department of Biochemistry and Molecular Pharmacology, New York University School of Medicine, New York, NY10016 USA; 15 CRCINA, Inserm, Université d’Angers, Université de Nantes, Nantes, France; 16 The Biodesign Institute, Center for Immunotherapy, Vaccines, and Virotherapy, Arizona State University, Tempe, AZ 85287-5401 USA; 17 Department of Microbiology and Immunology, University of Arkansas for Medical Sciences (UAMS), Little Rock, AR 72205 USA; 18 Department of Anatomy and Unit for Multidisciplinary Research in Biomedicine (UMIB), Institute of Biomedical Sciences Abel Salazar (ICBAS), University of Porto, Porto, Portugal; 19 Division of Infectious Diseases, Department of Medicine, Johns Hopkins University School of Medicine, Baltimore, MD USA; 20 The Public Health Research Institute (PHRI) at New Jersey Medical School, Rutgers Biomedical and Health Sciences (RBHS), Rutgers University, Newark, NJ USA; 21 Tubercolosis Research Section, Laboratory of Clinical Infectious Diseases, Division of Intramural Research, National Institute of Allergy and Infectious Diseases, National Institutes of Health, Bethesda, MD USA; 22 Institute of Infectious Disease and Molecular Medicine, Department of Clinical Laboratory Sciences, University of Cape Town, Cape Town, South Africa; 23 Laboratory of Immune System Biology, National Institute of Allergy and Infectious Diseases, National Institutes of Health, Bethesda, MD USA

**Correction to: Experimental & Molecular Medicine**

10.1038/s12276-018-0094-1 published online 22 May 2018

This article was originally published under a CC BY-NC-SA License, but has now been made available under a CC BY 4.0 License.

The PDF and HTML versions of the Article have been modified accordingly.

